# The Prognostic Value of CXCR4 in Ovarian Cancer: A Meta-Analysis

**DOI:** 10.1371/journal.pone.0092629

**Published:** 2014-03-21

**Authors:** Cheng-Fei Liu, Shu-Yan Liu, Xiao-Yun Min, Yuan-Yuan Ji, Na Wang, Dan Liu, Ning Ma, Zong-Fang Li, Ke Li

**Affiliations:** 1 Core Research Laboratory, The Second Affiliated Hospital, School of Medicine, Xi'an Jiaotong University, Xi'an, China; 2 Department of Reproductive Medicine, The First Affiliated Hospital, School of Medicine, Xi'an Jiaotong University, Xi'an, China; 3 Department of Rheumatology and Immunology, Rheumatology Institute, The Fifth Hospital of Xi'an, Xi'an, China; 4 Department of General Surgery, The Second Affiliated Hospital, School of Medicine, Xi'an Jiaotong University, Xi'an, China; Innsbruck Medical University, Austria

## Abstract

**Objective:**

Recent reports have shown that C-X-C chemokine receptor 4 (CXCR4) is expressed in ovarian cancer and plays an important role in metastasis. However, the prognostic value of CXCR4 in ovarian cancer remains controversial and has not been emphasized. The aim of this study is to evaluate the prognostic significance of CXCR4 in ovarian cancer by performing a meta-analysis.

**Methods:**

We systematically searched for studies evaluating the relationship between CXCR4 expression and the outcome of ovarian cancer patients. Only articles in which CXCR4 expression was detected by immunohistochemical staining were included. Hazard ratios (HRs) and relative risk (RR) with 95% confidence intervals (CIs) were pooled as effect size (ES) across studies for overall survival (OS) and progression-free survival (PFS).

**Results:**

A total of 729 patients from 7 studies (6 articles) were included in this meta-analysis. Our results showed that high CXCR4 expression was significantly associated with poor prognosis in terms of OS (ES, 2.81; 95% CI, 1.16–6.80; p = 0.022) and PFS (ES, 8.48; 95% CI, 2.13–33.70; p = 0.002) in ovarian cancer patients. The association between high CXCR4 expression and poor ovarian cancer prognosis in OS was also statistically significant in subgroups of Asian and III-IV patients constituting 70%.

**Conclusions:**

The present meta-analysis indicated that high CXCR4 expression was associated with poor prognosis in ovarian cancer. More studies, especially larger scale and well-matched researches, are warranted to clarify the prognostic effect of CXCR4 on the outcome of ovarian cancer.

## Introduction

Ovarian cancer is the fifth leading cause of cancer deaths occurring in women and leading cause of mortality from gynecologic cancer [1], with epithelial cancer being responsible for 90% of ovarian malignancies. Although ovarian cancer is among the most chemosensitive malignancies, and the currently established therapy of ovarian cancer includes radical surgical tumor debulking and subsequent platinum plus paclitaxel-based chemotherapy, the prognosis is still poor and the 5-year survival rate remains only 25% [2]. Hence, it is necessary to identify prognostic factors to predict the outcomes of patients, which could be effective in making strategies and improving survival for ovarian cancer. Traditional clinicopathological parameters, such as age, tumor histology, performance status and residual tumor volume are considered as independent predictors of prognosis in ovarian cancer [3], [4]. However, survivals vary even among patients who are in similar status and undergoing similar treatment. The underlying mechanism remains elusive. Identifying molecular biological prognostic factors could enable to predict patients' outcomes more accurately and provide novel therapeutic targets.

C-X-C chemokine receptor 4 (CXCR4) is a G-protein coupled chemokine receptor, exerts its biological effect by binding its ligand stromal cell-derived factor 1 (SDF-1, also called CXCL12), leading to alteration of cell skeleton rearrangement and cell migration [5]. It plays an important role in embryonic development [6], phagocytic cells migration [7] and differentiation [8]. Recent reports suggest that CXCR4 also plays a decisive role in tumor growth and metastasis. Directed metastasis of cancer cells is mediated by CXCR4 activation and migration of cancer cells towards CXCL12 expressing organs [9]–[11]. A study of the expression of 14 chemokine receptors showed that only CXCR4 was expressed within ovarian cancer cell lines [12]. Many studies indicated a close correlation exists between chemokine axis CXCL12/CXCR4 and ovarian cancer and recommend CXCR4 as an independent prognostic factor [13]–[16]. However, Popple et al and Pils et al showed that CXCR4 expression has no influence on survival in ovarian cancer [17], [18]. Insufficient samples and some other factors have resulted in controversial results of different clinical studies. The present meta-analysis aims to determine the value of CXCR4 as a prognostic marker for ovarian cancer.

## Materials and Methods

### Literature Search Strategy

We comprehensively searched PubMed, Cochrane library, CBM and EMBASE databases for relevant articles published until December 1st, 2013. Search terms included terms for Ovarian Cancer (“Ovarian Neoplasm”, “Ovarian Carcinoma”, “Ovarian Cancer”, “Ovarian Tumor”), CXCR4 (“CXCR4”, “Chemokine receptor 4”), Prognosis and Survival. In order to minimize the deviation caused during the search process, the reference lists of relative articles were also screened manually to further identify potential studies.

### Criteria for Inclusion and Exclusion

Studies eligible for inclusion in this meta-analysis met the following criteria in order to ensure the high quality of this article: (1) patients with distinctive ovarian cancer diagnosis by pathology; (2) CXCR4 expression was measured by immunohistochemistry (IHC) method; (3) full length paper with sufficient data on survival and CXCR4 expression. The following studies were excluded: (1) articles about cell lines or animals; (2) review articles without original data; (3) studies lacking information on survival.

### Data extraction and quality assessment

The following information were retrieved independently by 2 authors (CF Liu and SY Liu) from each publication: first author, publication year, country, number of patients enrolled, histology and disease stage, cut-off value, hazard ratio (HR) or relative risk (RR) and 95% confidence interval (CI) as well as the other related events. Inconsistencies in the research process were resolved through debate and consultations. If the above information were not mentioned in the original study, the item was treated as “Not Available (NA)”. The quality of the included studies was assessed by the Newcastle-Ottawa Scale (NOS). If a study did not clearly mention one of these key points, we considered that the point was not covered in the study, and the results may have underestimated the reported characteristics.

### Statistical analysis

HR or RR and 95% CI were used as the effective value to measure the impact of CXCR4 expression on survival of ovarian cancer patients in this meta-analysis. In the individual study, some of them provided HR or RR and 95% CI directly. If these statistical variables were not available in an article, we estimated from given data using methods reported by Tierney et al [19]. If a study provided both the results of multivariate analysis and univariate analysis, we chose the former. ES was calculated with Chi-squared tests according to Peto's method [20]. Heterogeneity test with inconsistency index (I^2^) statistic and Q statistic was performed. The I^2^ value was used to evaluate the heterogeneity (I^2^  = 0–50%, no or moderate heterogeneity; I^2^ >50%, significant heterogeneity). Fixed-effect model was used if there was no significant heterogeneity. Otherwise, the random-effect model was used. Egger's test, Begg's test and trim and fill method were performed to identify the possibility of publication bias [21], [22]. The robustness of the combined results was confirmed by sensitivity analysis in which the data of an individual study were removed each time. By convention, an observed ES >1 implied a poor survival for the group with increased CXCR4 expression. The impact of increased CXCR4 expression on survival was considered statistically significant if the 95% CI did not overlap with 1. All the p-values were two sided, and p<0.05 was considered statistically significant. All statistical analyses were conducted with STATA 12.0 (Stata Corporation, College Station, TX).

## Results

### Study selection and characteristics

**Figure 1:**
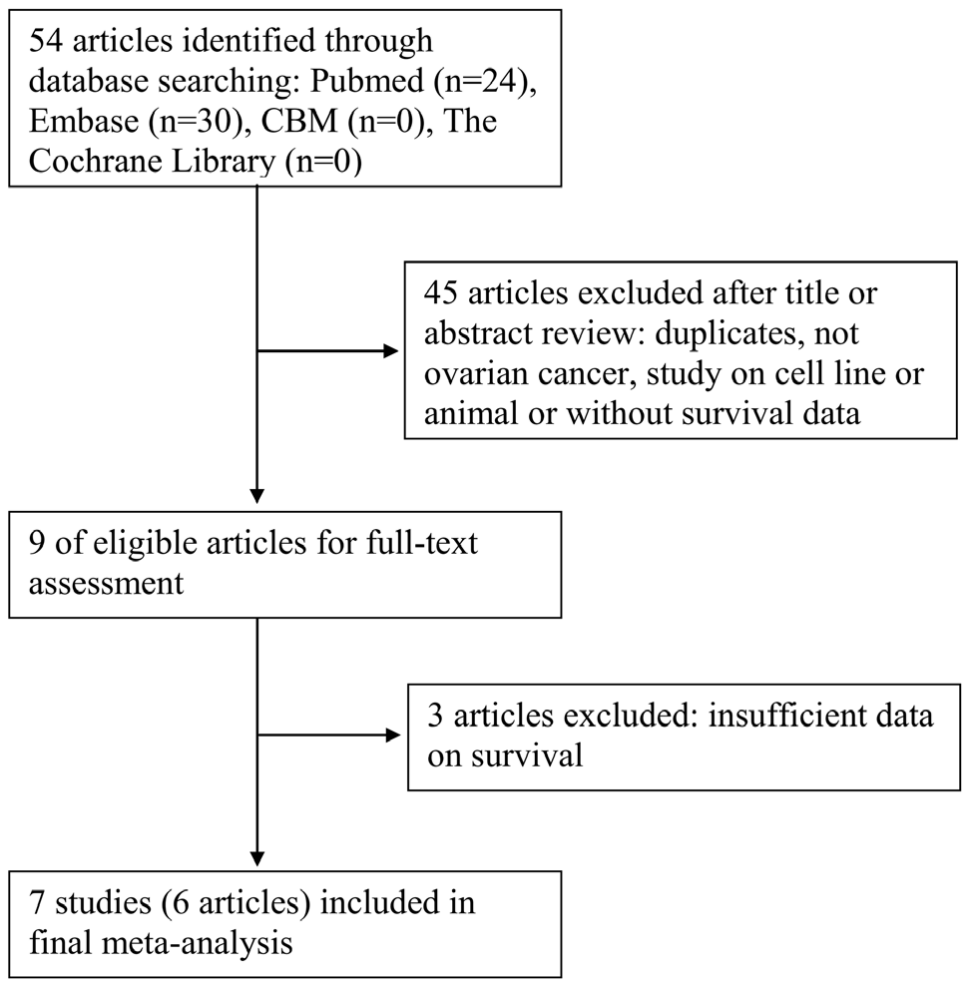
Flow chart of searching the relevant studies used in this meta-analysis.

As shown in Figure 1, a total of 54 articles were identified initially using the search strategy above. After reading the abstract and title, 45 papers were not applicable to our aim. The remaining 9 papers were approved through scrutinizing the entire paper. Among them, 3 articles were excluded because of insufficient data on survival. Finally, 7 studies (6 articles) were eligible for the present meta-analysis [13]–[18].

The main characteristics of the 7 studies were shown in Table 1. The studies were conducted in 4 countries (UK, China, Austria and Japan) and published between 2006 and 2013. The total number of patients included was 729, with sample sizes ranging from 44 to 241 patients. The median age ranged from 51 to 61 years old. The median follow-up period ranged from 26.1 to 167.0 months. Only 1 study defined the cut-off value by visible staining or not, while other studies used the complex score to define high CXCR4 expression. Among all of the included studies, HR or RR and 95% CI were obtained from the original articles directly in 4 studies. For the remaining studies, HRs and 95% CIs were calculated or extrapolated from Kaplan-Meier curves. On statistical method, all studies provided the results of multivariate analysis.

**Table 1:** Characteristics of the included studies.

First author	Year	Country	No. of Patients (CXCR4 High/Low)	Age (y)	Follow-up (m)	Histological type	FIGO stage	Chemotherapy regimens (n)	Residual tumor	Antibody	Subcellular localization	Cut-off	Survival	Analysis	NOS Score
Li	2013	China	124(75/49)	>55 y 53, ≤55 y 71	NA	S 52, M 38, E 15, U 19	I–II 31, III–IV 93	platinum-based chemotherapy 124	NA	Sigma	NA	Low 0–4; High 5–12 (IRS)	OS/PFS	Multi	7
Sekiya	2012	Japan	48(24/24)	52(27–69)	63.8(4.3–204.3)	C 48	I 24, II–IV 18 Unknown 6	platinum-based chemotherapy 37	Absent 38, Present 4	Chemicon	NA	Low 0–3; High 4–6 (IRS)	OS/PFS	Multi	9
Popple	2012	UK	241(214/27)	< 30 y 1, 30 y-60 y 92, >60 y 146	167.0(95.0–335.0)	S 128, E 30, M 33, C 18, U 31 O 11	I–II 89, III–IV 144, Unknown 8	Adjuvant therapy: Yes 168; No, 67	Absent 143, Present 201	R&D	Cytoplasm and nucleus (mainly nucleus)	Low 0–60; high 60–300 (H-score)	OS	Multi	8
Pils-cyt	2007	Austria	119(64/55)	58.6(27.6–87.2)	43.7(0.4–168.7)	S 65, E 23, M 6, C 6, U 12, Mixed 7	I–II 43, III–IV 76	CBP+PTX 81; CDDP+CTX 9	NA	R&D	Cytoplasm	Low 0–1; High 2–3	OS	Multi	8
Pils-nuc	2007	Austria	101(22/79)	59.2(47.1–71.3)	43.7(0.4–168.7)	S 55, E 20, M 6, C 5, U 11, Mixed 4	I–II 36, III–IV 65	CBP+PTX 81; CDDP+CTX 9	NA	R&D	nucleus	Low, no visible staining; High, visible staining	OS	Multi	8
Oda	2007	Japan	52(20/32)	58(36–77)	26.1(1.0–61.8)	S 49, E 4	I–II 7, III–IV 46	PTX+CBP 50; DOC+CBP 2	<2 cm 36, >2 cm 11	BD	NA	Low 0–1+ staining with <10% positive cells; High 2–3+ staining with >10% positive cells (IRS)	OS	Multi	6
Jiang	2006	China	44(26/18)	51(43–60)	37.0(7.0–70.0)	S 34, E 8, M 1, C 1	I–II 11, III–IV 33	CDDP+CTX 44	<1 cm 28, >1 cm 16	R&D	Cytoplasm and nucleus (mainly cytoplasm)	Low 0; High 1–12 (IRS)	OS/PFS	Multi	7

C, clear cell; S, serous; E, endometrioid; M, mucinous; U, Undifferentiated; O Others; NA, not available; FIGO, International Federation of Gynecology and Obstetrics; IRS, immunohistochemical score; OS, overall survival; PFS, progression-free survival; cyt, cytoplasm; nuc, nuclear; HR, Hazard Ratio; RR, Risk Ratio; Multi, multivariate analysis; NOS, Newcastle-Ottawa Scale; CDDP, Cisplatin; PTX, paclitaxel; CBP, carboplatin; CTX, cyclophosphamide; DOC, docetaxel.

### CXCR4 expression and prognosis of ovarian cancer

**Figure 2:**
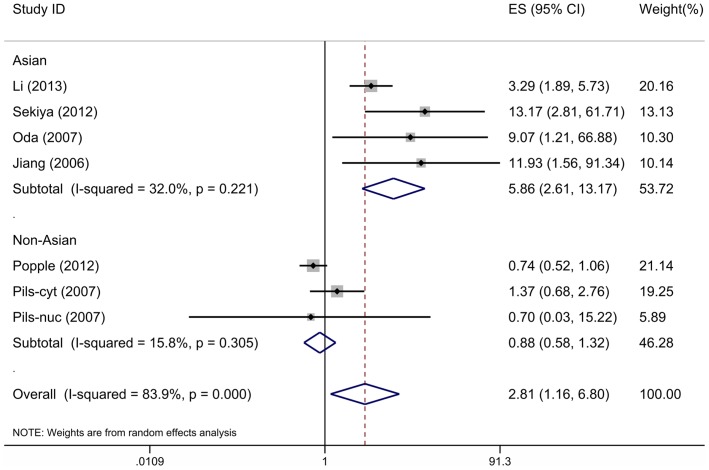
Forest plot illustrates the association between CXCR4 expression and OS of ovarian cancer.

7 studies investigating OS were pooled into the meta-analysis. As shown in Figure 2, high CXCR4 expression correlates with poor OS (ES, 2.81; 95% CI, 1.16–6.80; p = 0.022) with significant heterogeneity (I^2^ = 83.9%, p = 0.000). Table 2 shows the results of the main subgroup meta-analyses. When grouped by ethnicity, CXCR4 expression was significantly associated with poor OS in Asian patients (ES, 5.86; 95% CI, 2.61–13.17; p = 0.000) with less heterogeneity (I^2^  = 32.0%, p = 0.221), but not in non-Asian patients (ES, 0.88; 95% CI, 0.58–1.32; p = 0.529) with less heterogeneity (I^2^ = 15.8%, p = 0.305). The association between high CXCR4 expression and poor ovarian cancer prognosis in OS remained statistically significant in studies with more III–IV patients (ES, 4.13; 95% CI, 2.15–7.92; p = 0.000). However, not significant association between high CXCR4 expression and poor OS was found in studies when grouped according to subcellular localization or the median follow-up period.

**Table 2:** Associations between CXCR4 expression and OS of ovarian cancer grouped by selected factors.

Variables	No. of studies	No. of patients	ES (95% CI)	I^2^ (%)
Country				
Asian	4	268	5.86(2.61–13.17)	32.0
Non-Asian	3	461	0.88(0.58–1.32)	15.8
Stage III–IV (%)				
>70	3	220	4.13(2.15–7.92)	8.9
≤70	4	509	1.65(0.59–4.62)	78.7
Subcellular localization				
Cytoplasm	2	163	3.25(0.41–25.90)	74.3
Nucleus	2	342	0.74(0.52–1.05)	0.0
Follow-up (mo)				
>60	2	289	2.82(0.17–47.01)	92.1
≤60	4	316	3.12(0.83–11.72)	55.2

**Figure 3:**
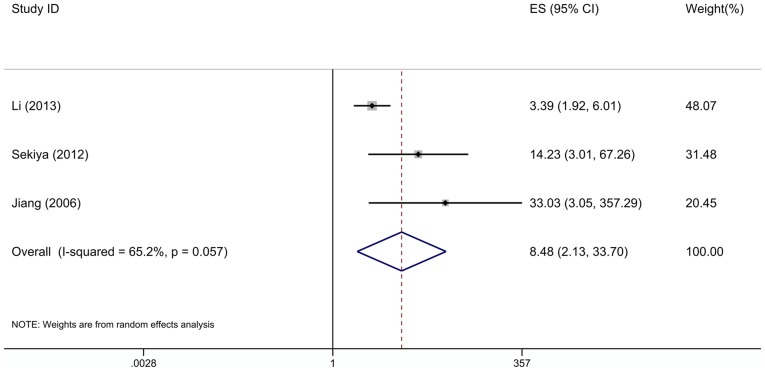
Forest plot showing the association between CXCR4 expression and PFS of ovarian cancer.

3 studies investigating PFS were pooled into the meta-analysis. As shown in Figure 3, high CXCR4 expression also predicts poor PFS (ES, 8.48; 95% CI, 2.13–33.70; p = 0.002) with significant heterogeneity (I^2^ = 65.2%, p = 0.057).

### Publication bias analysis

**Figure 4:**
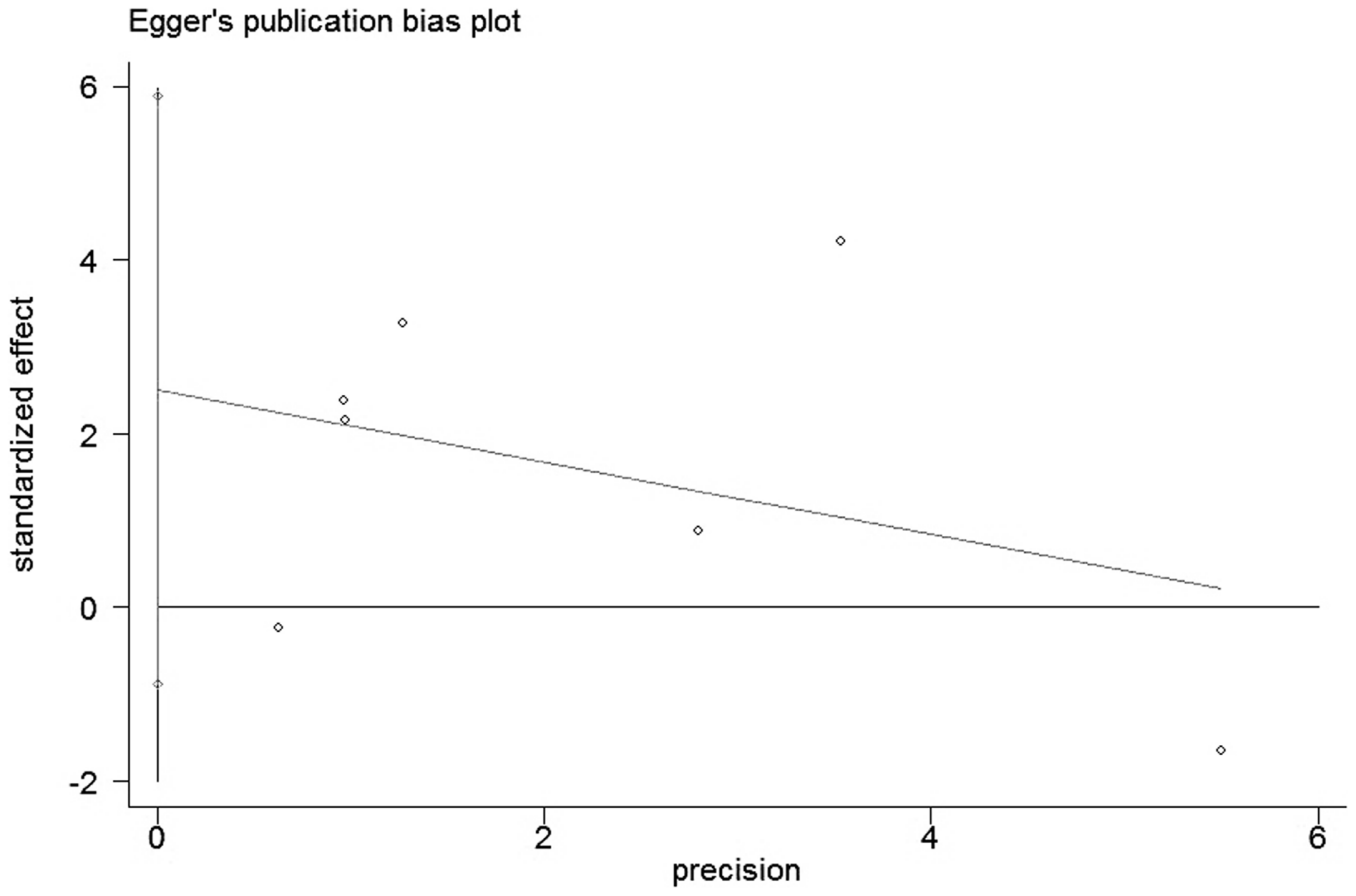
Egger's publication bias plot of the studies assessing CXCR4 expression and OS in ovarian cancer.

The Egger's publication bias plot presented no proof of obvious publication bias (Figure 4). There was no evidence of publication bias as suggested by Egger's and Begg's tests (Egger's test, p = 0.421; Begg's test, p = 1.000). Besides, trim and fill method showed that there is no significant change after trimming and filling, suggesting stable conclusions (data not shown).

### Sensitivity analysis

**Figure 5:**
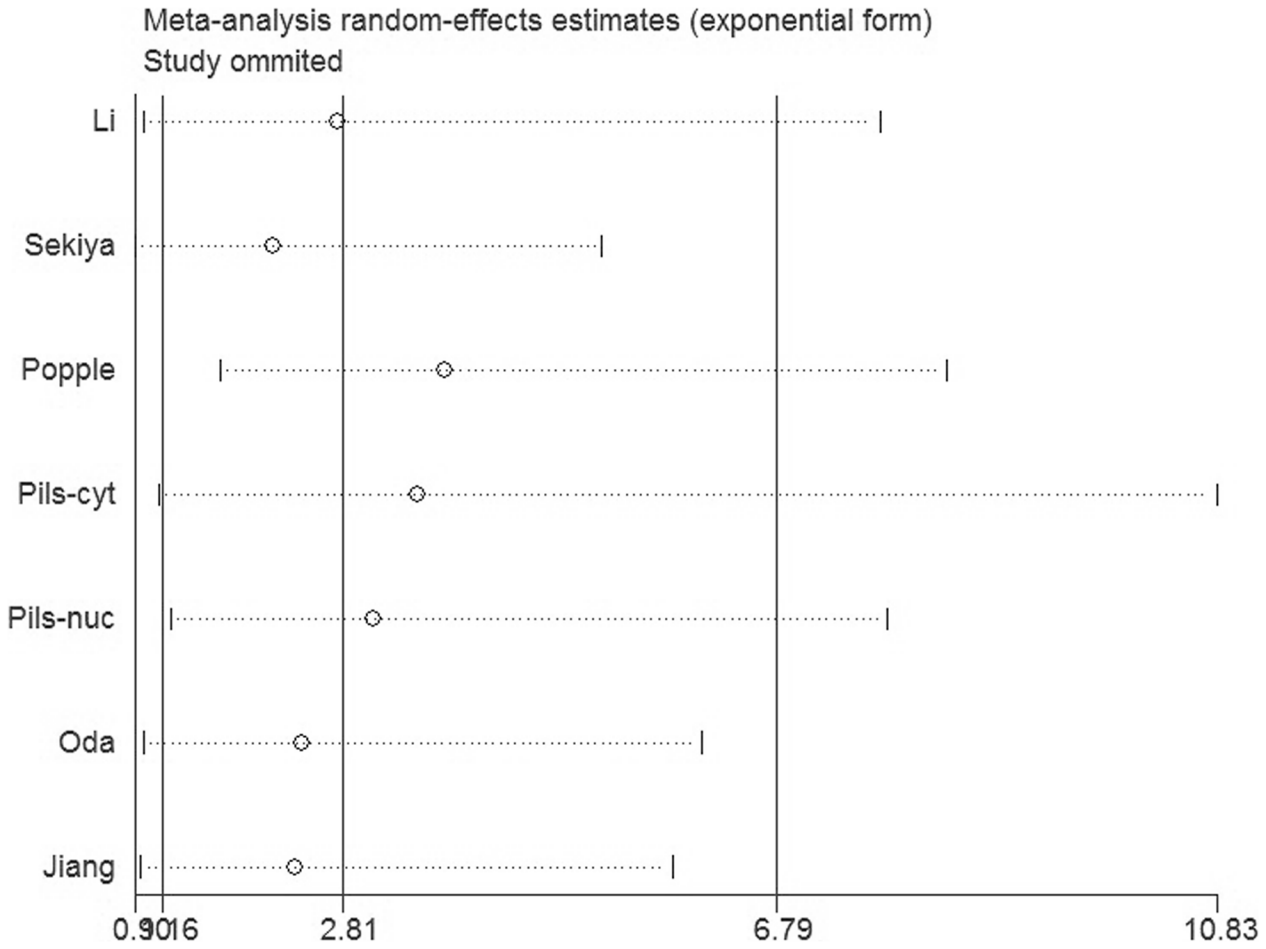
Sensitivity analysis of all the studies assessing OS.

In order to gauge results stability, a sensitivity analysis, in which one study was deleted at a time, was performed. The results were shown in Figure 5. Both of the corresponding pooled ES were not significantly changed, suggesting the robustness of our results.

## Discussion

Nowadays, factors which influence the prognosis of ovarian cancer patients are not completely understood. Survivals vary even among patients who are in similar status and undergoing similar treatment. There has been great interest in identifying molecular biological prognostic and predictive markers for patients with ovarian cancer as these markers can help predict patients' outcomes more accurately and provide novel therapeutic targets [23]–[25].

CXCR4 is a chemokine receptor predominantly expressed on lymphocytes where it actives chemotaxis. CXCL12 is the only physiological ligand for CXCR4. Many Studies demonstrated that CXCR4/CXCL12 is not only critical molecular determinant for events including maintaining embryo development, mediating immune and inflammatory reactions, the modulation of hematopoietic system, but also plays an important role in stimulating the metastatic process of many different neoplasm, where CXCR4 is able to activate a plethora of phenomena such as chemotaxis, invasion, angiogenesis and proliferation [26], [27]. Recent reports showed that high expression of CXCR4 indicates poor prognosis in patients with ovarian cancer [13]–[16], but others showed that no correlation was found between them [17], [18]. Different subcellular localization of CXCR4 and some other factors might have resulted in controversial results of different clinical studies. In previous reports, CXCR4 expression was found in nucleus, cytoplasm, and membrane of diverse cancer cells by means of immunohistochemistry. Salvucci et al found that only cytoplasmic staining of CXCR4 had significant impact on prognosis of breast cancer patients, but not nuclear staining [28]. Strong CXCR4 nuclear staining was associated with significantly better outcome in early-stage non-small-cell lung cancer (NSCLC) [29]. This meta-analysis aimed to examine the association between high CXCR4 expression and prognosis of ovarian cancer.

In this meta-analysis, we first evaluated the association of CXCR4 expression with OS and PFS. The pooled results demonstrated that high CXCR4 expression correlates with poor prognosis in terms of OS and PFS. A considerable degree of heterogeneity was noticed after the HR or RR were pooled in OS (I^2^ = 83.9%). Heterogeneity is an important factor affecting the quality of meta-analysis, subgroup analyses are essential. Our subgroup analyses showed that when grouped according to the countries of individual studies, the heterogeneity was significantly reduced, and the combined ES of Asian studies and non-Asian studies were 5.86 (95% CI: 2.61–13.17; I^2^ = 32.0%) and 0.88 (95% CI: 0.58–1.32; I^2^ = 15.8%), respectively. Asian patients with high levels of CXCR4 expression in cancer cells get remarkably poor prognosis, while non-Asians did not. It indicated that the ethnicity or geographic settings may contribute to the infection of CXCR4 expression on the prognosis of ovarian cancer. However, the number of studies and the number of patients included are all small, and there are also many confounding factors among these 7 studies. We must note these points when interpret the result. Besides, subgroup analyses according to the proportion of stage III-IV patients showed that high CXCR4 expression was also related significantly with poor prognosis in studies with more stage III–IV patients (pooled ES, 4.13; 95% CI: 2.15–7.92; I^2^ = 8.9%), suggesting that high expression of CXCR4 predicts poor prognosis in patients with advanced ovarian cancer. These results increase the likelihood that high CXCR4 expression is an independent risk factor for ovarian cancer.

Some limitations of this meta-analysis need to be acknowledged. First, mentioned above, the number of studies and patients included are very small. Results from our meta-analysis should be interpreted with caution. Second, heterogeneity was found in the main analysis. Meta regression was unable to be performed as the number of included studies was small. Heterogeneity may have arisen from the different characteristics of the subjects and the various histological types of ovarian cancer. However, the attempt to perform subgroup analysis by other important clinical factors in ovarian cancer patients such as TNM, histological types, differentiation and treatment were failed due to lack of sufficient data. Third, the follow-up times varied widely, from 0.4 to 204.3 months. Finally, the methodology for immunohistochemistry could affect the prognostic value due to the various detecting antibodies, subcellular localization of CXCR4 protein and the application of different cut-off values for determining high CXCR4 levels.

In conclusion, our meta-analysis showed that high CXCR4 expression was associated with poor prognosis in terms of OS and PFS in ovarian cancer. The outcome is much worse in Asian and advanced ovarian cancer patients. Assessing CXCR4 expression could provide better prognostic information for patients with ovarian cancer and be used as a novel therapeutic target. More studies, especially large scale, multi-center and well-matched cohort research, were warranted to clarify the prognostic effect of CXCR4 on the outcome of ovarian cancer.

## Supporting Information

Checklist S1 **PRISMA checklist.**
(DOC)

## References

[1] Siegel R, Naishadham D, Jemal A (2012) Cancer statistics, 2012. CA Cancer J Clin 62: 10–29. 22237781 10.3322/caac.20138

[2] Chiang YC, Chen CA, Chiang CJ, Hsu TH, Lin MC, et al (2013) Trends in incidence and survival outcome of epithelial ovarian cancer: 30-year national population-based registry in Taiwan. J Gynecol Oncol 24: 342–351. 24167670 10.3802/jgo.2013.24.4.342 PMC3805915

[3] Landrum LM, Java J, Mathews CA, Lanneau GS Jr, Copeland LJ, et al (2013) Prognostic factors for stage III epithelial ovarian cancer treated with intraperitoneal chemotherapy: a Gynecologic Oncology Group study. Gynecol Oncol 130: 12–18. 23578540 10.1016/j.ygyno.2013.04.001 PMC4870593

[4] Warner LL, Dowdy SC, Martin JR, Lemens MA, McGree ME, et al (2013) The impact of perioperative packed red blood cell transfusion on survival in epithelial ovarian cancer. Int J Gynecol Cancer 23: 1612–1619. 24172098 10.1097/01.IGC.0000436089.03581.6b PMC4306564

[5] Kucia M, Jankowski K, Reca R, Wysoczynski M, Bandura L, et al (2004) CXCR4-SDF-1 signalling, locomotion, chemotaxis and adhesion. J Mol Histol 35: 233–245. 15339043 10.1023/b:hijo.0000032355.66152.b8

[6] Nagasawa T, Hirota S, Tachibana K, Takakura N, Nishikawa S, et al (1996) Defects of B-cell lymphopoiesis and bone-marrow myelopoiesis in mice lacking the CXC chemokine PBSF/SDF-1. Nature 382: 635–638. 8757135 10.1038/382635a0

[7] Yamada M, Kubo H, Kobayashi S, Ishizawa K, He M, et al (2011) The increase in surface CXCR4 expression on lung extravascular neutrophils and its effects on neutrophils during endotoxin-induced lung injury. Cell Mol Immunol 8: 305–314. 21460863 10.1038/cmi.2011.8 PMC4002449

[8] Sanchez-Martin L, Estecha A, Samaniego R, Sanchez-Ramon S, Vega MA, et al (2011) The chemokine CXCL12 regulates monocyte-macrophage differentiation and RUNX3 expression. Blood 117: 88–97. 20930067 10.1182/blood-2009-12-258186

[9] De Falco V, Guarino V, Avilla E, Castellone MD, Salerno P, et al (2007) Biological role and potential therapeutic targeting of the chemokine receptor CXCR4 in undifferentiated thyroid cancer. Cancer Res 67: 11821–11829. 18089812 10.1158/0008-5472.CAN-07-0899

[10] Hassan S, Buchanan M, Jahan K, Aguilar-Mahecha A, Gaboury L, et al (2011) CXCR4 peptide antagonist inhibits primary breast tumor growth, metastasis and enhances the efficacy of anti-VEGF treatment or docetaxel in a transgenic mouse model. Int J Cancer 129: 225–232. 20830712 10.1002/ijc.25665

[11] Bartolome RA, Ferreiro S, Miquilena-Colina ME, Martinez-Prats L, Soto-Montenegro ML, et al (2009) The chemokine receptor CXCR4 and the metalloproteinase MT1-MMP are mutually required during melanoma metastasis to lungs. Am J Pathol 174: 602–612. 19147814 10.2353/ajpath.2009.080636 PMC2630568

[12] Scotton CJ, Wilson JL, Scott K, Stamp G, Wilbanks GD, et al (2002) Multiple actions of the chemokine CXCL12 on epithelial tumor cells in human ovarian cancer. Cancer Res 62: 5930–5938. 12384559

[13] Jiang YP, Wu XH, Shi B, Wu WX, Yin GR (2006) Expression of chemokine CXCL12 and its receptor CXCR4 in human epithelial ovarian cancer: an independent prognostic factor for tumor progression. Gynecol Oncol 103: 226–233. 16631235 10.1016/j.ygyno.2006.02.036

[14] Sekiya R, Kajiyama H, Sakai K, Umezu T, Mizuno M, et al (2012) Expression of CXCR4 indicates poor prognosis in patients with clear cell carcinoma of the ovary. Hum Pathol 43: 904–910. 22169254 10.1016/j.humpath.2011.08.002

[15] Oda Y, Ohishi Y, Basaki Y, Kobayashi H, Hirakawa T, et al (2007) Prognostic implications of the nuclear localization of Y-box-binding protein-1 and CXCR4 expression in ovarian cancer: their correlation with activated Akt, LRP/MVP and P-glycoprotein expression. Cancer Sci 98: 1020–1026. 17459055 10.1111/j.1349-7006.2007.00492.x PMC11159905

[16] Li J, Jiang K, Qiu X, Li M, Hao Q, et al.. (2013) Overexpression of CXCR4 is significantly associated with cisplatin-based chemotherapy resistance and can be a prognostic factor in epithelial ovarian cancer. BMB Rep: In press. 10.5483/BMBRep.2014.47.1.069 PMC4163846 24209634

[17] Popple A, Durrant LG, Spendlove I, Rolland P, Scott IV, et al (2012) The chemokine, CXCL12, is an independent predictor of poor survival in ovarian cancer. Br J Cancer 106: 1306–1313. 22415233 10.1038/bjc.2012.49 PMC3314783

[18] Pils D, Pinter A, Reibenwein J, Alfanz A, Horak P, et al (2007) In ovarian cancer the prognostic influence of HER2/neu is not dependent on the CXCR4/SDF-1 signalling pathway. Br J Cancer 96: 485–491. 17245339 10.1038/sj.bjc.6603581 PMC2360022

[19] Tierney JF, Stewart LA, Ghersi D, Burdett S, Sydes MR (2007) Practical methods for incorporating summary time-to-event data into meta-analysis. Trials 8: 16. 17555582 10.1186/1745-6215-8-16 PMC1920534

[20] Yusuf S, Peto R, Lewis J, Collins R, Sleight P (1985) Beta blockade during and after myocardial infarction: an overview of the randomized trials. Prog Cardiovasc Dis 27: 335–371. 2858114 10.1016/s0033-0620(85)80003-7

[21] Egger M, Davey Smith G, Schneider M, Minder C (1997) Bias in meta-analysis detected by a simple, graphical test. BMJ 315: 629–634. 9310563 10.1136/bmj.315.7109.629 PMC2127453

[22] Begg CB, Mazumdar M (1994) Operating characteristics of a rank correlation test for publication bias. Biometrics 50: 1088–1101. 7786990

[23] Skirnisdottir I, Bjersand K, Akerud H, Seidal T (2013) Napsin A as a marker of clear cell ovarian carcinoma. BMC Cancer 13: 524. 24191930 10.1186/1471-2407-13-524 PMC4228360

[24] Mhawech-Fauceglia P, Wang D, Menesses T, Chandavarkar U, Ough F, et al (2012) Pax-8 is a reliable marker in making the diagnosis in advanced stage epithelial ovarian carcinoma and primary peritoneal carcinoma for neoadjuvant chemotherapy on cell block and biopsy specimens. Histopathology 60: 1019–1020. 22348438 10.1111/j.1365-2559.2011.04172.x

[25] Monk BJ, Kaye SB, Poveda A, Herzog TJ, Aracil M, et al (2014) Nibrin is a marker of clinical outcome in patients with advanced serous ovarian cancer treated in the phase III OVA-301 trial. Gynecol Oncol 132: 176–180. 24211400 10.1016/j.ygyno.2013.10.032

[26] Murphy PM (2001) Chemokines and the molecular basis of cancer metastasis. N Engl J Med 345: 833–835. 11556308 10.1056/NEJM200109133451113

[27] Shayegi N, Meyer C, Lambert K, Ehninger G, Brand M, et al (2014) CXCR4 blockade and Sphingosine-1-phosphate activation facilitate engraftment of haematopoietic stem and progenitor cells in a non-myeloablative transplant model. Br J Haematol 164: 409–413. 24180707 10.1111/bjh.12641

[28] Salvucci O, Bouchard A, Baccarelli A, Deschenes J, Sauter G, et al (2006) The role of CXCR4 receptor expression in breast cancer: a large tissue microarray study. Breast Cancer Res Treat 97: 275–283. 16344916 10.1007/s10549-005-9121-8

[29] Spano JP, Andre F, Morat L, Sabatier L, Besse B, et al (2004) Chemokine receptor CXCR4 and early-stage non-small cell lung cancer: pattern of expression and correlation with outcome. Ann Oncol 15: 613–617. 15033669 10.1093/annonc/mdh136

